# Preclinical Evaluation of Novel Conserved *Neisseria gonorrhoeae* Vaccine Candidates Identified Through Infection-Informed Antigen Discovery

**DOI:** 10.3390/vaccines14090798

**Published:** 2026-09-10

**Authors:** Shea K. Roe, Bo Zheng, Sunita Gulati, Sanjay Ram, Paola Massari

**Affiliations:** 1 Department of Immunology, Tufts University School of Medicine, Boston, MA 02111, USA; shea.roe@tufts.edu; 2 Division of Infectious Diseases and Immunology, University of Massachusetts Chan Medical School, Worcester, MA 01605, USA; bo.zheng@umassmed.edu (B.Z.); sunita.gulati@umassmed.edu (S.G.); sanjay.ram@umassmed.edu (S.R.)

**Keywords:** *Neisseria gonorrhoeae*, antigens, bactericidal antibodies, protection

## Abstract

**Background:** The continued emergence of antimicrobial-resistant *Neisseria gonorrhoeae* highlights the urgent need for an effective vaccine. We evaluated the immunogenicity and vaccine potential of six new gonococcal conserved hypothetical proteins identified through our immunobioinformatics-based pipeline (Candidate Antigen Selection Strategy, CASS). **Methods:** NGO0588, NGO0694, NGO0757, NGO0861, NGO1438 and NGO1802 proteins were expressed in *E. coli*, purified, and used to immunize mice, adjuvanted with Alum and MPLA. Immunological analyses included quantitative and qualitative evaluation of antigen-specific antibody responses, cytokines, serum bactericidal activity (SBA), and protective efficacy in the mouse gonococcal vaginal colonization model. **Results:** The six antigens elicited robust antigen-specific IgG and IgM antibody responses against *N. gonorrhoeae*. Antisera to NGO0588, NGO0861, NGO1430 and NGO1802 were bactericidal against different gonococcal strains. IgG antibodies recognizing the antigens were detected in sera obtained from women with disseminated gonococcal infection, particularly against NGO1802, supporting immunogenicity in humans. Taking immunological and biochemical properties into consideration, NGO0861, NGO1438 and NGO1802 were combined in a trivalent vaccine formulation. The trivalent vaccine elicited robust antigen-specific and *N. gonorrhoeae*-reactive serum IgG and IgM, mucosal IgG, and higher SBA titers than each individual antigen. However, in the mouse gonococcal vaginal colonization model, vaccinated mice and adjuvant control mice did not show statistically significant differences in time to infection clearance or bacterial burdens. **Conclusions:** A multiantigen vaccine composed of NGO0861, NGO1438 and NGO1802 elicited antibodies that were functional in vitro but did not lead to protection in vivo. Immunological analyses in vitro and in vivo emphasize the importance of evaluating antigen and antibody quality and functional activity for vaccine target prioritization of next-generation gonococcal multicomponent vaccines.

## 1. Introduction

*Neisseria gonorrhoeae*, the etiologic agent of the sexually transmitted infection (STI) gonorrhea, is a major global public health threat. The World Health Organization estimates that more than 80 million new gonococcal infections occur annually worldwide, making gonorrhea the second-most prevalent bacterial sexually transmitted infection after chlamydia. More than 540,000 cases were reported in the U.S in 2024 [1,2]. In women, infection is frequently asymptomatic and can lead to severe reproductive tract sequelae, including pelvic inflammatory disease, ectopic pregnancy, infertility, and adverse pregnancy outcomes. Gonococcal co-infections with *Chlamydia* and syphilis are frequent, and gonorrhea increases susceptibility to and transmission of human immunodeficiency virus (HIV) [3], further amplifying the public health impact of this STI. Despite ongoing efforts to improve prevention and treatment, gonorrhea incidence continues to increase in some geographical areas worldwide, along with emergence and global dissemination of antimicrobial-resistant strains. The gonococcus has developed resistance to every class of antibiotics previously used for treatment (i.e., sulfonamides, penicillins, tetracyclines, macrolides, and fluoroquinolones) and recently has shown reduced susceptibility or resistance to extended-spectrum cephalosporins worldwide, decreasing the effectiveness of current first-line therapies [4,5]. Antimicrobial-resistant *N. gonorrhoeae* is now designated a high-priority pathogen and there is a strong need for new strategies to control gonorrhea transmission, morbidity and infection management [6,7]. Development of an effective vaccine is considered the most sustainable long-term solution for gonorrhea control, but currently there is no licensed vaccine against gonorrhea, despite decades of investigation. Challenges to vaccine development include including antigenic and phase variation, molecular mimicry of host structures, complement resistance, active modulation of innate and adaptive immune responses, and the lack of naturally acquired protective immunity in humans [8,9,10,11,12].

Retrospective epidemiological studies noted reduced gonorrhea incidence among recipients of the outer membrane vesicle (OMV)-based serogroup B meningococcal vaccines, MeNZB and Bexsero^®^ (also called 4CmenB), which contain detergent-extracted OMVs plus three recombinant antigens (fHbp, NHBA and NadA [13]) [12,14,15,16,17]. Sera from mice immunized with 4CMenB showed cross-reactivity with multiple gonococcal antigens and bactericidal antibody responses against *N. gonorrhoeae,* accelerated gonococcal clearance and lower gonococcal bacterial burdens in a mouse model of gonococcal vaginal colonization [18,19,20]. However, recent prospective human clinical trials have failed to show efficacy of 4CMenB against gonorrhea in men who have sex with men (MSM), a population at high risk for STIs [21,22]. Gonococcal OMVs have also been evaluated in mouse studies, where bactericidal antibodies and accelerated clearance have been shown in a mouse vaginal colonization model (IL-12 microencapsulated OMV vaccine [23], GonoVac [24]). However, a Phase 2 trial evaluating gonococcal native OMVs was terminated because pre-defined efficacy criteria were not met [25,26,27]. Given the limited success of OMV-based vaccines, gonococcal subunit vaccines represent viable alternatives, and evidence of protection in a mouse model of gonococcal vaginal colonization has been shown for some antigens [28,29,30,31,32]. Proteins that are shared with *N. meningitidis* may also be attractive from the perspective of acceptability and marketing [33,34].

Our previous analysis of the gonococcal transcriptome during natural human mucosal infection indicated substantial differences in gonococcal gene expression profiles compared to bacteria growth in vitro [35,36]. This suggested that proteins important during infection may have been overlooked as vaccine targets identified by conventional discovery approaches in vitro, highlighting the importance of incorporating infection-associated gene expression data into antigen selection. We developed an infection-informed antigen discovery platform (Candidate Antigen Selection Strategy, CASS) by integrating transcriptomic and immunobioinformatic analyses and identified several uncharacterized proteins as potential vaccine targets [37]. Initial studies showed that three proteins among these targets, NGO0690, NGO0948 and NGO1701, led to accelerated bacterial clearance and reduced gonococcal burden in a mouse genital tract infection model when used as a multi-component vaccine [38,39]. NGO1701 was recently characterized as Csp, a periplasmic copper-storage protein involved in metal homeostasis, adding biological relevance to its protective potential [40]. Building on these findings, we sought to expand the repertoire of CASS targets by evaluating a second set of infection-associated antigens, NGO0588, NGO0694, NGO0757, NGO0861, NGO1438, and NGO1802. In the present study, we investigated their immunogenicity and protective efficacy in support of continued advancement of multicomponent vaccines targeting conserved gonococcal proteins expressed during natural human infection.

## 2. Materials and Methods

### 2.1. Antigens

The antigens utilized were NGO0588, a DUF4189 domain-containing protein (WP_003691328) (UniProt accession number Q5F918); NGO0694, a predicted autotransporter outer membrane β-barrel domain-containing protein (WP_229683582.1) (UniProt accession number Q5F8R8); NGO0757, a predicted periplasmic protein and Spy/CpxP family protein refolding chaperone (WP_003688677.1) (UniProt accession number Q5F8L5); NGO0861, a hypothetical lipoprotein (WP_020996828.1) (UniProt accession number Q5F8C4); NGO1438, a predicted DsbC family protein, Thiol:disulfide interchange protein (WP_003689299.1) (UniProt accession number Q5F6V7); and NGO1802, a predicted OmpH family outer membrane protein (WP_157147426.1) (Uniprot accession number Q5F5W7).

### 2.2. Gene and Protein Sequence Analysis, and Protein Structure Predictions

Gene sequence presence and conservation in *N. gonorrhoeae* and *N. meningitidis* were evaluated using the PubMLST database [41], including evaluation of allele presence and representation, and gene polymorphisms. Protein structure predictions were obtained with AlphaFold (v.6) [42,43] based on the available protein sequences in the NCBI Reference Sequence: NC_002946. Structure modeling was rendered with PyMol (v. 3.1.4) [44].

### 2.3. Cloning, Expression and Purification of Recombinant Proteins

Cloning, expression and protein purification were outsourced to GenScript (Piscataway, NJ, USA). Briefly, using primers designed based on the available protein sequences of *N. gonorrhoeae* FA1090 in NCBI (NC_002946.2), C-terminal 6× His-tagged truncated NGO0588, NGO0757, NGO0861, NGO1438 and NGO1802 and full-length NGO0694, were cloned in a pET30a plasmid containing a kanamycin resistance cassette using NdeI and HindIII restriction sites. Protein expression was induced in transformed *E. coli* BL21 Star (DE3) with 0.5 mM IPTG. Bacterial cultures were lysed with 50 mM Tris-HCl and 150 mM NaCl buffer, pH 8.0, and supernatants containing soluble proteins NGO0757, NGO0861, NGO1438 and NGO1802 were directly applied onto a Ni^++^ agarose resin and eluted with a 20–500 mM Imidazole gradient in buffer containing 50 mM Tris-HCl, 500 mM NaCl and 1% Triton X-100, pH 8.0. Inclusion bodies containing insoluble proteins NGO0588 and NGO0694 were resuspended in 50 mM Tris-HCl and 7 M guanidine hydrochloride buffer, pH 8.0, and purified in denaturing conditions with buffer containing 8 M urea. Column fractions were examined by SDS-PAGE/Coomassie staining to assess purity. Protein-containing fractions were pooled and dialyzed against PBS pH 7.4 containing 10% glycerol (NGO0757, NGO0861, NGO1438 and NGO1802) and 0.5 M L-Arginine (NGO0588 and NGO0694). The protein concentration was measured by the bicinchoninic acid assay (BCA) (Thermo Fisher Scientific, Waltham, MA, USA). Gel imaging was done using a Bio-Rad ChemiDoc Gel Imaging System with Image Lab Software (Hercules, CA, USA).

### 2.4. Immunization of Mice

Female BALB/c mice (6 weeks old) (Jackson Labs, Bar Harbor, ME, USA) were housed, cared for, and immunized according to NIH, Tufts University and University of Massachusetts Chan Medical School IACUC-approved protocols (#B2024-11 and #PROTO202000072, respectively). Mice were immunized subcutaneously three times at 2-weeks or 3weeks apart with the purified proteins individually (10 µg/mouse/dose) using Alum Adjuvant (G-Biosciences #7861215) (St. Louis, MO, USA) (1:1 *v*/*v* ratio with the antigen) + Monophosphoryl Lipid A (MPLA) (Avanti Lipids, Alabaster, AL, USA); 10 µg/mouse/dose) as adjuvants. For studies with the antigen combination vaccine (NGO0861, NGO1438 and NGO1802) (henceforth referred to as 3-Ag vaccine), proteins were used at 10 µg/mouse/dose as above, or, for the high-dose studies, 10 µg/mouse/dose of NGO0861 and 25 µg/mouse/dose of NGO1438 and NGO1802. Control mice were immunized with Alum + MPLA alone. Pre-immune (Pr) sera were collected prior to the first immunization, immune sera two weeks after each immunization (1st, 2nd, and 3rd), and vaginal lavages two weeks after the last immunization. All sera and lavages were stored at −80 °C until use.

### 2.5. Bacterial Strains and Growth Conditions

*N. gonorrhoeae* F62 (Pil+/Opa+) and FA1090 strains were grown overnight on solid medium (GC agar plates supplemented with 1% IsoVitaleX or on chocolate agar plates) at 37 °C in a 5% CO_2_ incubator, or in liquid GCB broth supplemented with 1% IsoVitaleX. Bacterial suspensions (O.D._600nm_ = 1, corresponding to 1–2 × 10^9^ bacteria/mL) were diluted to the desired concentrations. For some experiments, bacteria suspensions were killed with 1% paraformaldehyde for 1 h at 4 °C, washed and resuspended in PBS.

### 2.6. Dot Blot Analysis

Purified proteins (100 ng in 5 µL) and *N. gonorrhoeae* F62 or FA1090 (1.5 µg total protein content in 5 µL) were spotted on nitrocellulose filters. Membranes were blocked with 5% milk in PBS/Tween 20 (PBS-T), incubated overnight at 4 °C with pooled mouse immune sera (1:1000 dilution), followed by anti-mouse IgG secondary AP-conjugated antibody (Southern Biotech, Birmingham, AL, USA) for detection of immunoreactive dots with NBT/BCIP (5-bromo-4-chloro-3-indolyl phosphate/Nitroblue Tetrazolium) chromogenic substrate (Bio-Rad) and imaging with a Bio-Rad ChemiDoc Gel Imaging System.

### 2.7. Antibody ELISA

ELISA plates (Immulon 4 HBX) were coated with purified proteins (2 μg/mL) or formalin-fixed *N. gonorrhoeae* (1–1.5 × 10^8^ bacteria/mL), as previously described [38]. Plates were blocked and incubated with serial dilutions of pooled mouse sera or vaginal lavages, and antibody production was evaluated using AP-conjugated secondary anti-mouse total IgG, IgG1, IgG2a and IgM antibodies (Southern Biotech), followed by 1-step PNPP (p-nitrophenyl phosphate) reagent (Thermo Fisher Scientific) and spectrophotometric detection at O.D._405 nm._ Sera and vaginal lavages were tested in triplicate or quadruplicate. Quantification of antibody production (µg/mL) was carried out using antibody reference standard curves (Southern Biotech) and a linear regression function, and the Th2/Th1 ratio was calculated as IgG2a/IgG1 (µg/mL) [37]. IgG antibodies in human sera were measured in by incubating ELISA plates with banked, de-identified sera (1:100 dilution) obtained from women with disseminated gonococcal infection (DGI) [45] (*n* = 7). These sera belonged to a samples collection obtained from Dr. Peter Rice, MD, University of Massachusetts Chan Medical School [38]; their use was approved by the University of Massachusetts Chan Medical School IRB, and their collection and use was previously approved by the Institutional Review Board (IRB) of (at that time) the Trustee of Health and Hospitals of the City of Boston; subjects provided informed consent. Their use for the current study was determined not to constitute human subject research and therefore did not require IRB approval at Tufts University. Each individual serum sample was tested in triplicate; IgG levels were determined as O.D._405_ values and expressed as the mean O.D._405_ minus that of the control antigen without serum (blank) ± SD. The pooled whole normal human serum (NHS) control was from Pel-Freez Biologicals (#34019, Rogers, AK, USA).

### 2.8. Cytokine ELISA

For measurement of serum IL-4, IL-10, IL-12p70, IFN-γ, IL-6 and TNF-α levels, pooled mouse sera were examined by ELISA with Opt-EIA kits (BD Biosciences, San Jose, CA, USA), and for IL-1 β, an ELISA MAX kit (BioLegend. San Diego, CA, USA) was used, per the manufacturer’s specifications. Sera were tested in triplicate or quadruplicate. Cytokines were expressed in pg/mL ± SD.

### 2.9. Serum Bactericidal Activity (SBA)

*N. gonorrhoeae* F62 and FA1090 were grown as above and resuspended at 2–4 × 10^4^ CFU/mL in HBSS supplemented with CaCl_2_ (0.15 mM), MgCl_2_ (1 mM) and 2% BSA. Bacterial suspensions were incubated with serially diluted heat-inactivated pooled mouse sera for 20 min at room temperature, followed by addition of IgG/IgM-depleted pooled normal human serum (Pel-Freez Biologicals) (10% *v*/*v* for F62 or 20% (*v*/*v*) for FA1090) as a complement source. Aliquots of the suspensions were plated immediately on GC agar plates in triplicate (Time 0), and the remaining suspension was incubated for 30 min at 37 °C followed by plating of new aliquots (Time 30). After incubation of the plates overnight at 37 °C in a 5% CO_2_ incubator, bacterial survival was determined by counting colony-forming units (CFUs) at T0 and T30, and expressed as percentage (T30/T0) ± SD. The highest serum dilutions that yielded ≤50% survival after 30 min were considered as the bactericidal titers. Bacteria alone and bacteria incubated with complement alone were used as controls Sera were tested in triplicate or quadruplicate.

### 2.10. Mouse Model of Gonococcal Vaginal Challenge

Female BALB/c mice (*n* = 20) were immunized as described above. Two weeks after the last immunization, mice were evaluated to select those in the diestrus phase of the estrous cycle (*n* = 10). Premarin (Pfizer, New York, NY, USA)) (0.5 mg in 200 µL water) was administered subcutaneously at −2, 0, and +2 days relative to the challenge (Day 0) to prolong the estrus phase and to increase susceptibility to *N. gonorrhoeae* infection [46]. A combination of vancomycin, trimethoprim and streptomycin (VTS) was used for controlling the mouse vaginal microflora without affecting *N. gonorrhoeae* survival. *N. gonorrhoeae* FA1090 was used to challenge mice intravaginally (2.7 × 10^7^–3.1 × 10^7^ colony forming units, CFUs), and infection burden was monitored daily. Vaginal swabs were collected, eluted with 100 µL of normal saline and serially diluted for plating onto chocolate agar plates containing VTS, colistin and neomycin. Plates were incubated for 24 h at 37 °C in a 5% CO_2_ incubator and gonococcal colonies were enumerated to calculate CFUs.

### 2.11. Statistical Analysis

Statistical significance was determined with GraphPad Prism 11.0.2 (GraphPad Software, Inc., San Diego, CA, USA) using unpaired *t* test, one-way analyses of variance (ANOVA) with Tukey’s or Dunnett’s multiple comparisons test, and 2-way ANOVA with Tukey’s multiple comparisons test. Statistically significant *p* values are reported in the figure legends. For mouse challenge experiments, the median time to bacterial clearance in the mouse challenge model was determined using Kaplan–Meier survival curves, and compared between groups using the Mantel–Cox log-rank test. Bacterial burden over time (cumulative infection) was computed for each mouse as the mean area under the curve (AUC) of the log_10_ CFU vs. time and groups were compared using the Mann–Whitney non-parametric test [29].

## 3. Results

### 3.1. Bioinformatics Analysis of the Six Hypothetical Proteins from the CASS Pool

Details about the NGO0588, NGO0694, NGO0757, NGO0861, NGO1438 and NGO1802 proteins are shown in Table 1. The criteria and the bioinformatics tools used for the CASS design were previously described in detail [37].

Gene sequence presence and conservation were examined in the gonococcal genomes and meningococcal genomes available in the PubMLST database [41] (Table 2 and Table S1, respectively). Only the alleles present in >5% of strains are listed. The six genes were present in all gonococcal strains and were part of the gonococcal core genome (Ng cgMLST v2.0).

NGO0588 (NEIS1253), encoded by the *ngo0588* gene (NGO_RS02970), is a hypothetical protein of 160 amino acids with a predicted signal peptide cleavage site between amino acids 20 and 21, and sequence homology to a DUF4189 domain-containing protein in the pfam13827 family proteins with six well-conserved cysteines. NGO0588 is predicted to be localized in the periplasm and/or outer membrane, and its expression may be regulated by NrrF [47]. *ngo0588* is represented by 80 *N. gonorrhoeae* alleles (Table 2), with a total of 78 polymorphic sites; no non-synonymous mutations are seen in the most frequent allele (allele 10), which is present in >50% of strains (20,050 out of the 36,931 total strains). The meningococcal homologue of *ngo0588*, called *nmb1317a*, encodes for an uracil-xanthine permease family protein involved in membrane transport, which has high genetic variability in the most represented allele (allele 4) (Table S1). The NMB1317a protein is found in the outer membrane vesicle protein content of the meningococcal vaccine [48].

NGO0694 (NEIS2653), encoded by the *ngo0694* gene (NGO_RS03485), is a 914 amino acid-long protein with sequence homology to an autotransporter outer membrane β-barrel domain-containing protein, Ata-3, and, in its N-terminal region, to an adhesin from nontypeable *H. influenzae* [49]. *ngo0694* is represented by 171 alleles, and has over 500 polymorphic sites, but none are in highly frequent alleles. The most frequent allele (allele 2) has an internal stop codon mutation that may truncate the protein at residue 214, and less represented alleles contain a frameshift mutation that may lead to loss of residues 231–311 and 317–399 (~55% of the total isolates in PubMLST) (Table 2). No alleles could be assigned for ~9% of strains due to incomplete sequence data, as genes were located at the end of contigs (Table 2, NV). NGO0694 is not present in *N. meningitidis*.

NGO0757 (NEIS1146) is a 144 amino acid-long hypothetical periplasmic protein, with a predicted signal peptide cleavage site between amino acids 29 and 30, and sequence homology to a Spy/CpxP family protein refolding chaperone [50]. Analysis of *ngo0757* (NGO_RS03805) indicated a total of 63 alleles, with 144 polymorphic sites and no non-synonymous mutations in the most represented allele (allele 21) (Table 2). Similarly, analysis of the meningococcal homolog of *ngo0757* did not show non-synonymous mutations in the most frequent alleles (1 and 8) (Table S1), supporting gene sequence conservation.

NGO0861 (NEIS2662) is encoded by the *ngo0861* gene (NGO_RS04260) as a 61 amino acid-long hypothetical lipoprotein with a signal peptide cleavage site between amino acids 18 and 19. This protein is predicted to be localized the periplasm or the outer membrane, and has been previously reported present in the gonococcal envelope and in membrane vesicles that are naturally released by gonococci [51]. *ngo0861* was the most conserved gene (*p*-distance = 0.00007), with 50 alleles and 49 polymorphic sites, none of which localized in the most represented allele (allele 1) (90% frequency) (Table 2). This gene was also absent in *N. meningitidis*.

NGO1438 (NEIS0490) is a putative DsbC family protein member, also annotated as Thiol:disulfide interchange protein, encoded by the *ngo1438* gene (NGO_RS07170). Composed of 261 amino acids and containing a predicted signal peptide cleavage site between residues 19 and 20, NGO1438 is predicted to be localized in the periplasm, and was detected in both gonococcal envelopes and membrane vesicles [51]. *ngo1438* is represented by 154 alleles, with 242 polymorphic sites in alleles other than the most frequent alleles (6 and 13) (Table 2). Its meningococcal homolog is *nmb0550*, with 579 polymorphic sites and several non-synonymous mutations in highly represented alleles (Table S1), indicating some sequence diversity. The NMB0550 protein is also found in meningococcal outer membrane vesicles [52].

NGO1802 (NEIS0172), annotated as a putative member of the OmpH/Skp family, is a 166 amino acid-long protein with a predicted signal peptide cleavage site between residues 23 and 24. Its cellular localization is predicted to be periplasmic, possibly membrane-associated (inner or outer membrane), and it is reported present in the gonococcal envelope [51]. *ngo1802* (NGO_RS08995) is represented by 122 alleles, with 73 polymorphic sites and a frequent isoleucine-to-threonine non-synonymous mutation in position 2 (Table 2), being the most diverse gene (*p*-distance = 0.0048) among the six targets. Its meningococcal homolog is *nmb0181*, with over 600 alleles and 380 polymorphic sites but only one non-synonymous mutation in a less represented allele (Table S1). NMB0181 is thought to contribute to the protective effect of 4CMenB [53].

### 3.2. Protein Purification and Structure Prediction

**Figure 1:**
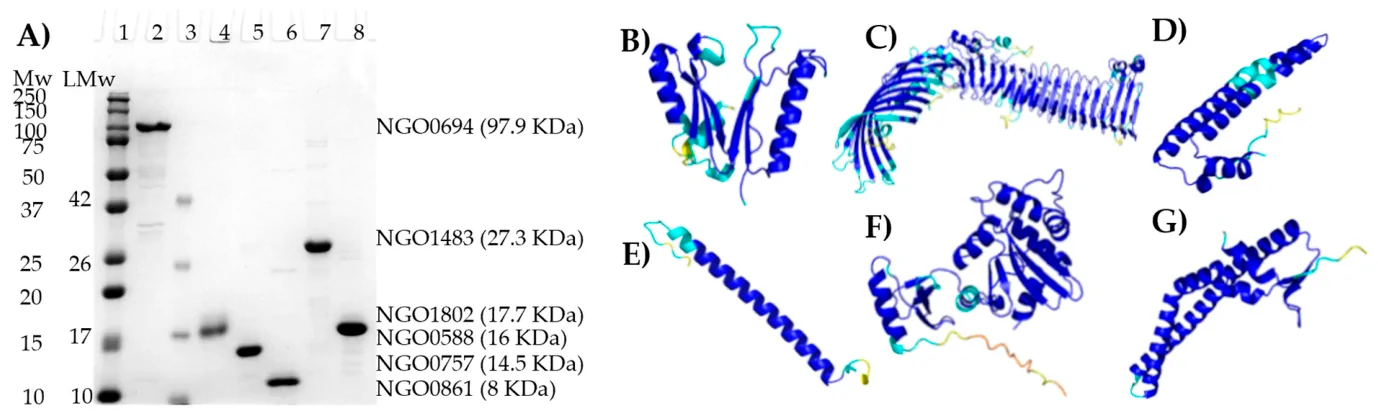
Structure analysis of the CASS antigens. (**A**) SDS-PAGE and Coomassie staining of the purified recombinant proteins. Lane 1, Molecular weight marker (Mw); Lane 2, NGO0694; Lane 3, Low-range molecular weight marker (LMw); Lane 4, NGO0588; Lane 5, NGO0757; Lane 6, NGO0861; Lane 7, NGO1438; Lane 8, NGO1802. (**B**) Cartoon model (side view) of the predicted structures of NGO0588, (**C**) NGO0694, (**D**) NGO757, (**E**) NGO0861, (**F**) NGO1438 and (**G**) NGO1802, modeled with Alpha Fold (v.6) and PyMol (v 3.1.4) [43,44,45] and colored by confidence (dark blue, highest to orange/yellow, lowest).

His-tagged NGO0757, NGO0861, NGO1438 and NGO1802 were expressed recombinantly as soluble proteins and purified by Ni^++^ affinity chromatography in non-denaturing conditions; His-tagged NGO0588 and NGO0694 were expressed in inclusion bodies and purified in denaturing conditions. Column eluates were examined by Western blotting with a mouse anti-His antibody, and pooled positive fractions were dialyzed against PBS. L-Arginine (0.5 M) was added to facilitate refolding of NGO0588 and NGO0694. Purity was assessed by SDS-PAGE and Coomassie staining based on the predicted molecular weight of each protein (Figure 1A). The predicted structure of each antigen was modeled using Alpha Fold [42,43] and PyMol [45] (Figure 1B–G).

### 3.3. Analysis of Immune Responses to the Individual Antigens

**Figure 2:**
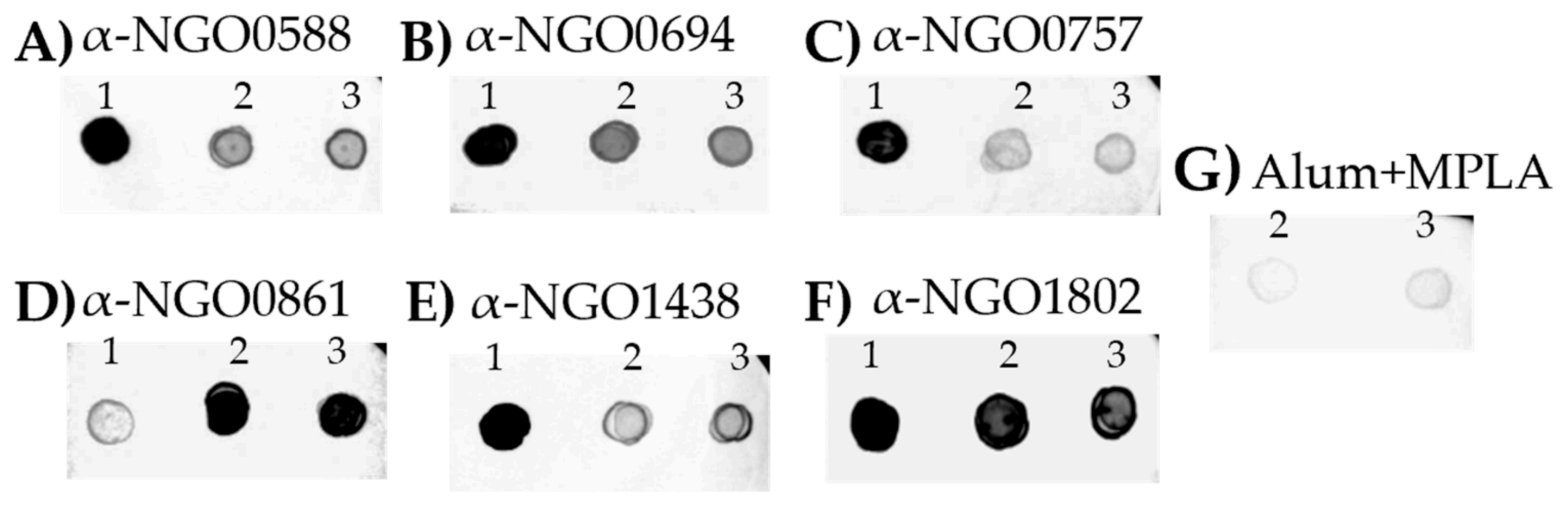
Dot blot. (**A**–**F**) Dot 1, purified proteins (100 ng in 5 µL); (**A**–**G**) Dots 2 and 3, *N. gonorrhoeae* F62 or FA1090, respectively (~1.5 µg total protein content in 5 µL). Each dot blot was incubated with pooled sera from mice immunized with the corresponding antigen and Alum + MPLA, or Alum + MPLA alone (**G**) (1:1000 dilution).

The purified proteins (10 µg each) were used to immunize female BALB/c mice with Alum + MPLA as adjuvants. As a control, a separate group of mice were immunized with the adjuvants alone. Qualitative analysis of the immunized mice sera by dot blot showed presence of IgG antibodies against the corresponding purified protein (Figure 2A–F, dots 1), with NGO0861 appearing a weaker immunogen (Figure 2D). Antisera were also tested against whole *N. gonorrhoeae* F62 (Figure 2A–F, dots 2) and FA1090 (Figure 2A–F, dots 3). Interestingly, despite the low immunoreactivity of purified NGO0861, this antigen was strongly recognized by the anti-NGO0861 mouse sera in both gonococcal strains (Figure 2D). In contrast, the anti-NGO0757 sera reacted strongly against the purified protein but only weakly against *N. gonorrhoeae* organisms (Figure 2C). The anti-NGO1802 mouse sera reacted strongly with both purified protein and gonococci (Figure 2F). Sera from mice immunized with Alum + MPLA showed low cross-reactivity with both bacterial strains (Figure 2G).

**Figure 3:**
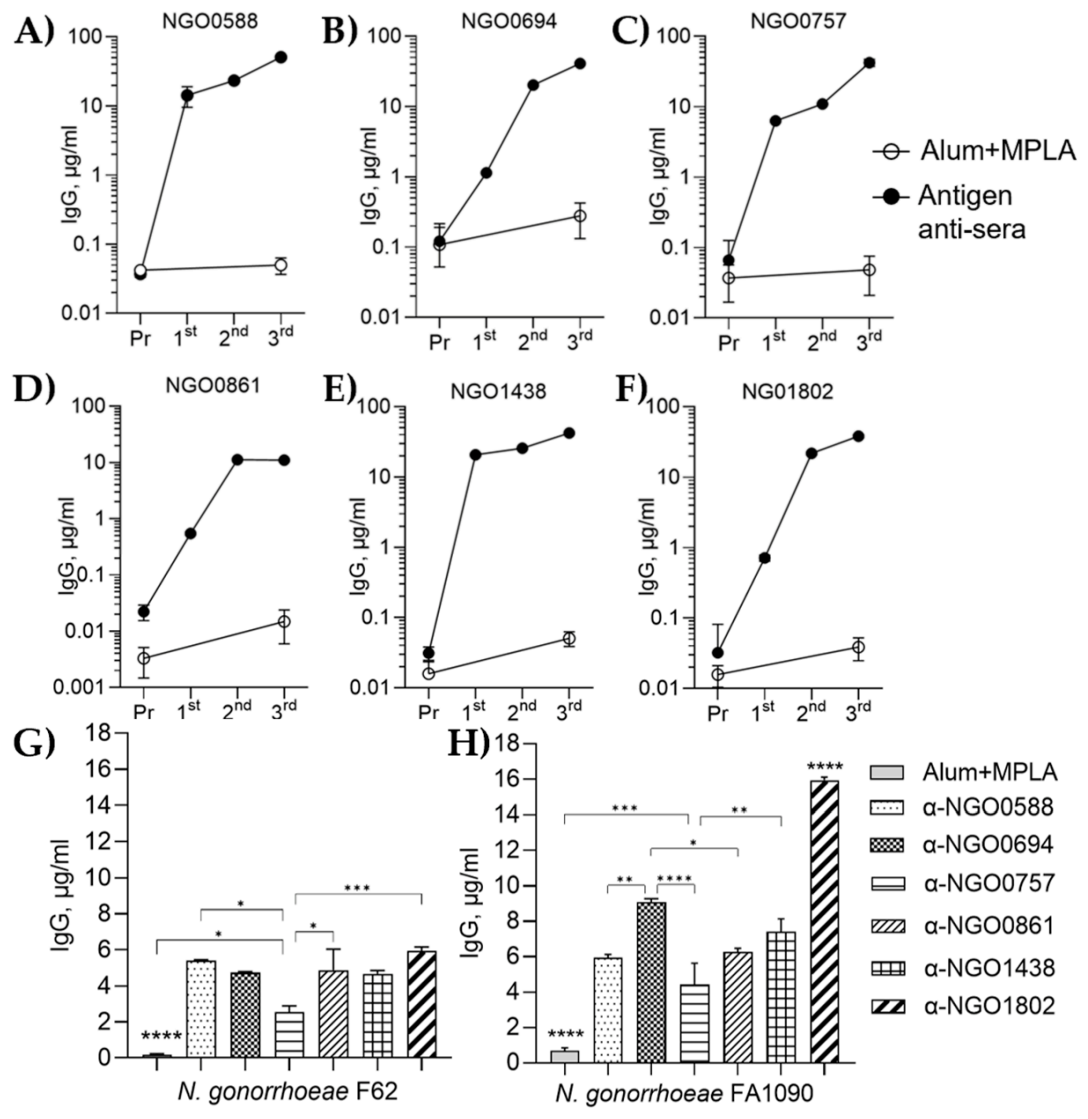
Total mouse serum IgG antibody ELISA. (**A**–**F**) IgG antibodies against the purified antigens in pooled sera from mice immunized with Alum + MPLA alone (open circles) or with the individual antigens and Alum + MPLA (closed circles). Pr: preimmune sera. 1st, 2nd and 3rd: sera collected 2 weeks after each immunization. Antibodies were quantified in µg/mL ± SD using a mouse IgG standard curve. (**G**,**H**) IgG antibodies in pooled sera (3rd) as above, against *N. gonorrhoeae* F62 and FA1090. * *p* = 0.02; ** *p* = 0.002; *** *p* < 0.0005 and **** *p* < 0.0001 by one way ANOVA with Tukey’s multiple comparisons test.

Next, antigen-specific serum antibody levels were quantified by ELISA. Overall, all antigens induced a strong IgG antibody response (Figure 3A–F), with NGO0861 remaining the weakest immunogen (Figure 3D). All antisera also recognized *N. gonorrhoeae* organisms in a whole-cell ELISA (Figure 3G,H), with anti-NGO0757 remaining a weaker responder serum (Figure 3G,H, dashed bars), as seen by dot blot. The adjuvant–control group did not mount a significant antibody response to the antigens (Figure 3A–F, open circles) or to the bacteria (Figure 3G,H, gray bars). The potential differences in the immunogenicity and immunoreactivity of the antigens may be due to intrinsic properties of the purified proteins, or, in the context of whole bacteria, expression levels and/or accessibility to antibodies for predicted periplasmic proteins (NGO0757) vs. predicted periplasmic/outer membrane proteins (NGO0861, NGO1438, and NGO1802).

Analysis of the IgG antibody subclasses showed significantly higher IgG2a levels than IgG1 for NGO0588 (Figure S1A), significantly higher IgG1 than IgG2a levels for NGO0694, NGO0757, NGO0861 and NGO1802 (Figure S1B, C, D and F, respectively), and relatively similar IgG subclass levels for NGO1438 (Figure S1E); NGO0588 induced the highest IgM levels (Figure S1G). Serum cytokine profiles also varied across antigens: NGO0588 induced high levels of IL-12p70 (Figure S2A) and NGO0861 and NGO1802 elicited higher IFN-γ production (Figure S2B), while IL-4 and IL-10 levels were comparable across all antigens (Figure S2C,D). Differences in inflammatory cytokines were also observed, with NGO0694 inducing significantly higher levels of IL-6 (Figure S2E), NGO1438 inducing higher TNF-α (Figure S2F), and NGO0588 high IL-1β (Figure S2G). These results also supported different immunological properties of the individual antigens.

The functions of elicited antibodies were evaluated by the serum bactericidal assay, where the serum bactericidal activity (SBA) of each mouse antisera was compared to that of the adjuvant control sera. As shown in Table 3, the highest-killing titers were reported for anti-NGO0588 against both *N. gonorrhoeae* F62 and FA1090. Anti-NGO0694 killed F62 (a serum-sensitive strain) but failed to kill FA1090 at the highest serum concentration tested (1/10); anti-NGO0757 did not kill F62 at the highest concentration used (1/10) and was not tested against FA1090. The latter two antigens were therefore considered low priority. Anti-NGO0861, anti-NGO1438 and anti-NGO1802 showed SBA titers between 1/20 and 1/40 against F62, and of 1/10 against FA1090 (Table 3). Despite NGO0588 inducing the highest SBA titers, the insoluble nature of this protein would be a significant hurdle for purification in a properly folded form at scale. Ultimately, NGO0861, NGO1430 and NGO1802 were considered the most attractive candidates.

**Figure 4:**
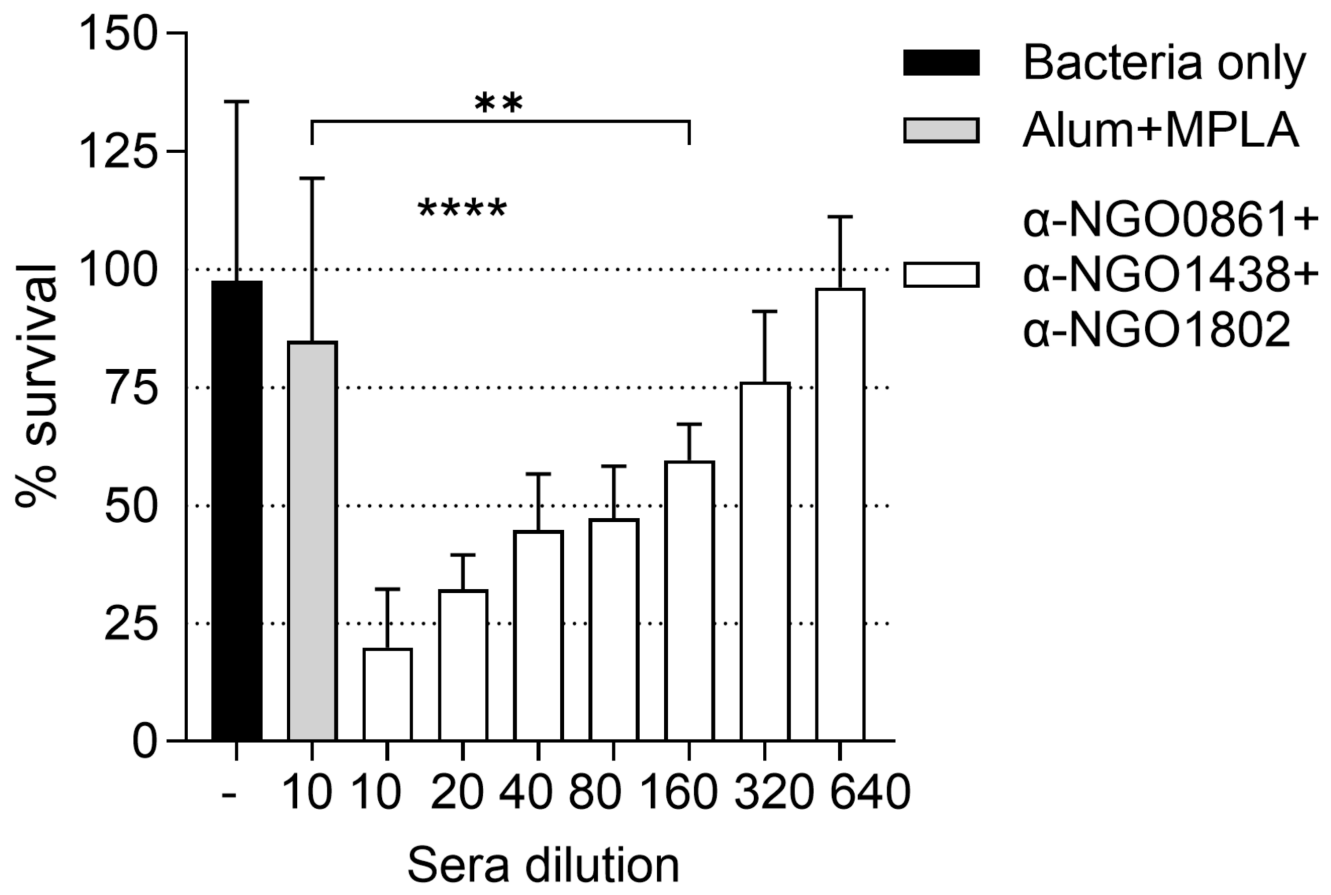
Serum bactericidal activity (SBA). *N. gonorrhoeae* F62 killing by pooled anti-NGO0861 + anti-NGO1438 + anti-NGO1802 mouse serum (1:1:1 *v*/*v*) (white bars) or Alum + MPLA alone serum (gray bars). Bacteria alone, black bars. Bacterial survival was expressed as % CFU at T30/T0 ± SD. Sera dilutions are indicated on the *x*-axis. **, *p* < 0.005 and ****, *p* < 0.0001 by one-way ANOVA with Dunnett’s multiple comparisons test vs. Alum + MPLA.

We previously observed that combining antisera against individual antigens synergistically increased SBA titers [38]. Similarly, mixing anti-NGO0861, anti-NGO1438 and anti-NGO1802 sera in equal amounts by volume led to higher SBA compared to each individual antiserum (Figure 4), supporting an additive effect.

Immunorecognition of the six targets by human sera from women with disseminated gonococcal infection (DGI) (*n* = 7) was also examined by ELISA. While uncomplicated mucosal gonococcal infections only induce modest gonococcal-specific antibodies [54], these levels tend to be higher in subjects with invasive forms of the disease, such as DGI [55]. Higher IgG antibody levels against NGO1802 were detected in the DGI sera than against the other antigens (Figure S3A), and, as expected, serum reactivity against whole gonococci was observed. IgG levels against NGO0694, NGO0861 and NGO1438 were slightly, but not statistically significantly, higher than those detected in commercially available normal human serum (NHS) control (Figure S3B). The latter also showed some reactivity against NGO1802 and *N. gonorrhoeae* F62 as well, likely due to the presence of preexisting cross-reactive antibodies elicited by prior colonization with commensal *Neisseriae* or *N. meningitidis* [56]. Based on these collective observations, a three-antigen combination vaccine composed of NGO0861, NGO1438 and NGO1802 was explored in vivo.

### 3.4. Analysis of Immune Responses to a Three-Antigen Combination Vaccine

**Figure 5:**
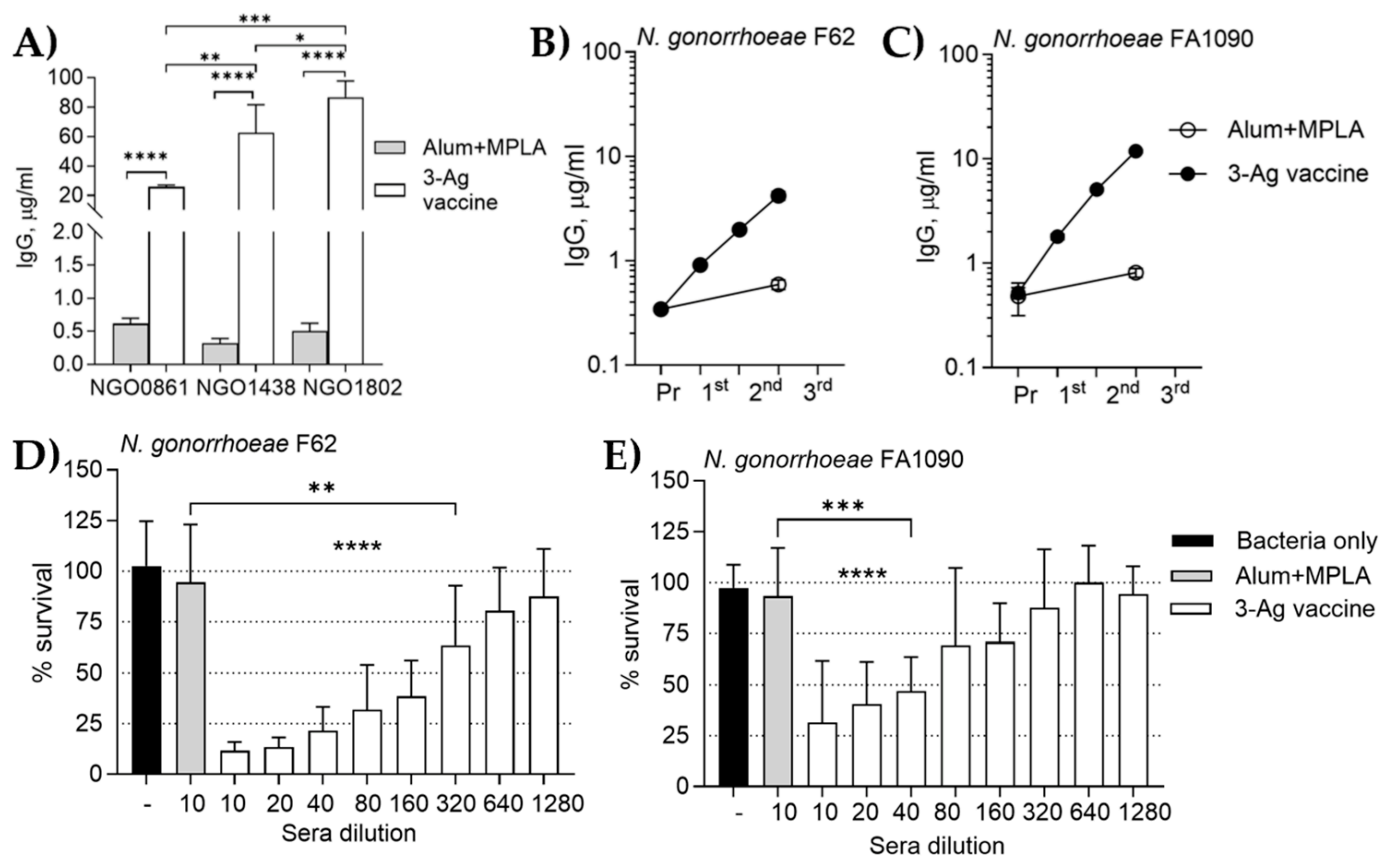
Total mouse serum IgG antibody ELISA and SBA titers. (**A**) IgG antibodies against the purified antigens in pooled sera (3rd) from mice immunized with Alum + MPLA alone (gray bars) or with the 3-Ag vaccine and Alum + MPLA (white bars). IgG antibodies were quantified in µg/mL ± SD using a mouse IgG standard curve. *, *p* = 0.03; **, *p* = 0.008, ***, *p* = 0.0002 by one-way ANOVA with Tukey’s multiple comparisons test. ****, *p* < 0.0001 by unpaired *t* test. (**B**,**C**) IgG antibodies in pooled sera as above against *N. gonorrhoeae* F62 and *N. gonorrhoeae* FA109. Alum + MPLA, open circles; 3-Ag vaccine, black circles. Pr: preimmune sera; 1st, 2nd and 3rd: sera collected 2 weeks after each immunization. (**D**) SBA of pooled sera from mice immunized with the 3-Ag vaccine (white bars) or with Alum + MPLA alone (gray bars) against *N. gonorrhoeae* F62 and (**E**) *N. gonorrhoeae* FA1090. Bacteria alone, black bars. Bacterial survival was expressed as % CFU at T30/T0 ± SD. Sera dilutions are indicated on the *x*-axis. **, *p* = 0.002, ***, *p* = 0.0002 and ****, *p* < 0.0001 by one-way ANOVA with Dunnett’s multiple comparisons test vs. Alum + MPLA.

A trivalent vaccine formulation, composed of NGO0861, NGO1438 and NGO1802 (10 µg/dose each at a 1:1:1 ratio) and Alum + MPLA (referred to as 3-Ag vaccine), was used to evaluate immune responses and protection against gonococcal vaginal challenge. A robust IgG antibody response against each antigen and against *N. gonorrhoeae* (Figure 5A–C) was confirmed by ELISA and was Th2-biased (IgG2a/IgG1 ratio < 1). Anti-gonococcal IgG antibody levels in vaginal lavages from the 3-Ag vaccine-immunized mice were also measured (Table S2A), and mirrored those induced by immunization with the individual antigens (Table S2B). IgM antibody levels against *N. gonorrhoeae* induced by the 3-Ag vaccine were overall slightly elevated compared to those induced by immunization with the individual antigens (Table S2A and B, respectively), but neither were statistically significantly different from the respective adjuvant controls. The serum cytokine profile was also similar to that elicited by the three individual antigens (see Figure S2), with comparable levels of IL-12p70 (1550 pg/mL ± 200), IL-4 (400 pg/mL ± 236), IL-10 (890 pg/mL ± 350), IL-6 and TNF-α (250 pg/mL ± 74 and 180 pg/mL ± 60, respectively), except for a decrease in IFN-γ production in response to the 3-Ag vaccine (1550 pg/mL ± 347). Antibody functional analysis showed an increase in SBA titers to up to 1/160 against *N. gonorrhoeae* F62 (Figure 5D), in agreement with the additive effect of the combination of individual sera (see Figure 4), and 1/40 against FA1090 (Figure 5E).

**Figure 6:**
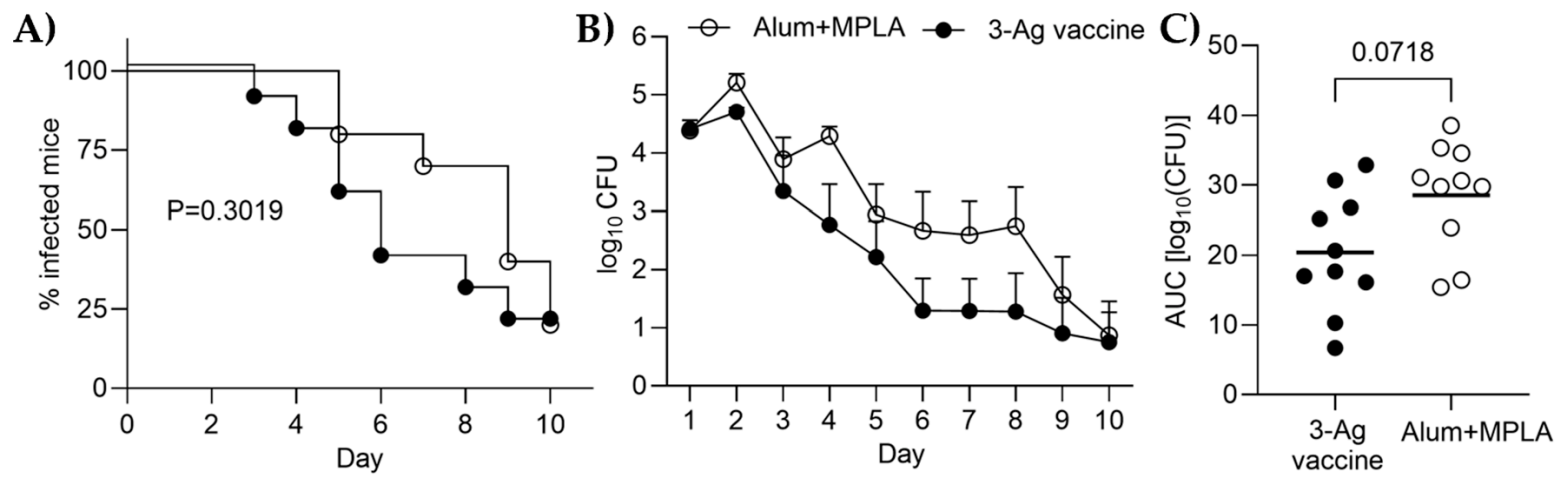
Mouse model of gonococcal vaginal colonization. Female BALB/c mice (*n* = 20 per group) immunized with the 3-Ag vaccine (10 µg each antigen) and Alum + MPLA (closed circles) or with Alum + MPLA alone (open circles) were challenged (*n* = 10 per group) intravaginally with *N. gonorrhoeae* FA1090 (2.6 × 10^7^ CFU). Vaginal swabs were collected daily and plated to enumerate gonococcal CFUs. (**A**) Time-to-clearance Kaplan–Meier curves (*p* determined by Mantel–Cox analysis); (**B**) Bacterial burden (log_10_ CFU ± SEM); (**C**) Area under the curve (AUC) (means ± 95% confidence intervals compared across groups (*p* determined by Mann–Whitney non-parametric test).

Two weeks after the last immunization, mice in the diestrus stage of the estrous cycle were infected vaginally with *N. gonorrhoeae* FA1090. The time to clearance of 50% of mice was 6 days for the vaccine group and 9 days for the adjuvant control group, but these differences were not statistically significant (Figure 6A). Similarly, there were no statistically significant differences observed when the bacterial burdens over time and the cumulative bacterial burden (area under the curve, AUC) among the groups were compared (Figure 6B,C).

### 3.5. Immune Responses to a High-Dose Three-Antigen Combination Vaccine

**Figure 7:**
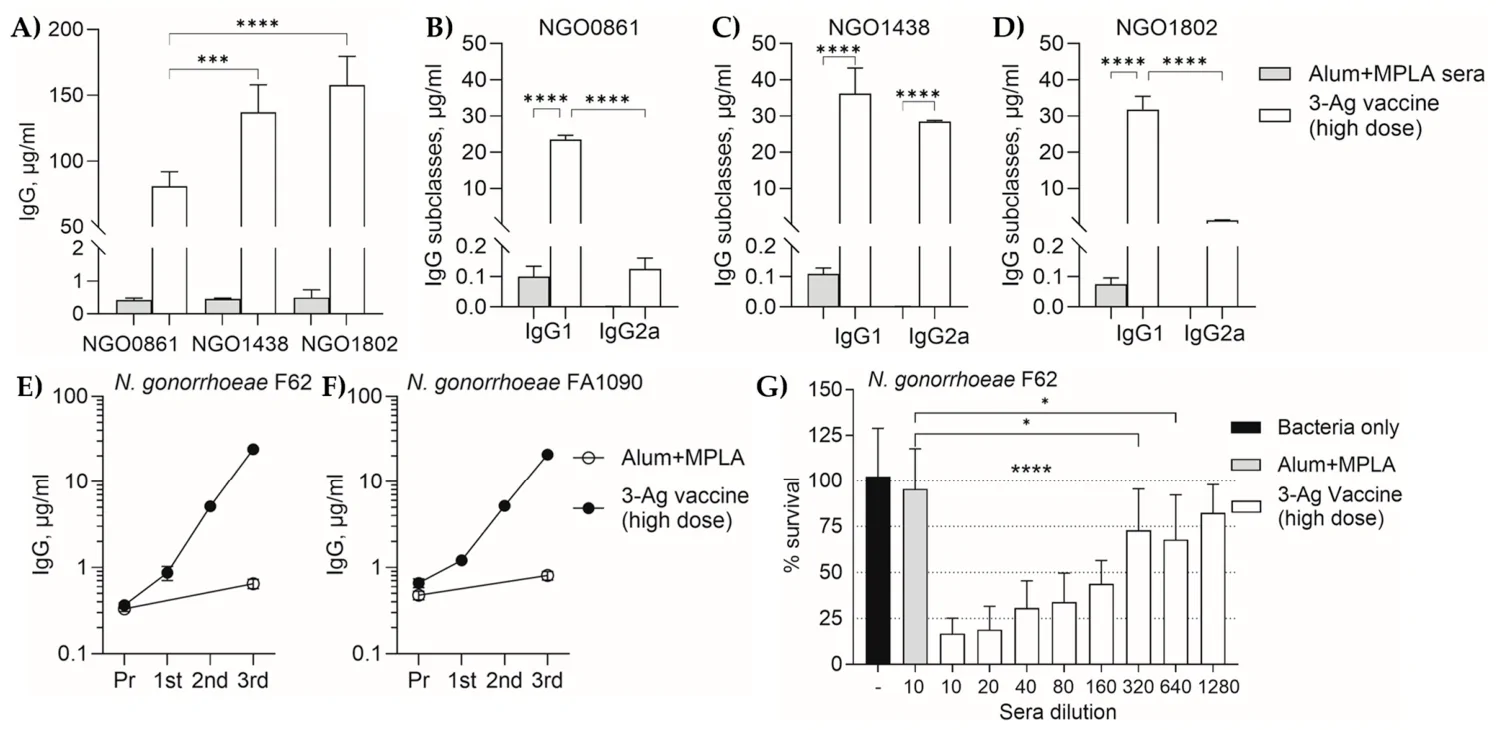
Total IgG antibody ELISA and SBA titers. (**A**) Total IgG antibodies and (**B**–**D**) IgG antibody subclasses against purified NGO0861, NGO1438 and NGO1802 in pooled sera (3rd) from mice immunized with Alum + MPLA alone (gray bars) or with the high-dose 3-Ag vaccine and Alum + MPLA (white bars). IgG antibodies were quantified in µg/mL ± SD using a mouse IgG standard curve. ***, *p* = 0.0003 and ****, *p* < 0.0001 by one-way ANOVA with Tukey’s multiple comparisons test. (**E**,**F**) Total IgG antibodies against *N. gonorrhoeae* F62 and *N. gonorrhoeae* FA1090, as above. High-dose 3-Ag vaccine, black circles; Alum + MPLA alone sera, open circles. Pr: preimmune sera. 1st, 2nd and 3rd: sera collected 2 weeks after each immunization. (**G**) SBA against *N. gonorrhoeae* F62. High-dose 3-Ag vaccine, white bars; Alum + MPLA alone, gray bars. Bacteria alone, black bars. Bacterial survival was expressed as % CFU at T30/T0 ± SD. Sera dilutions are indicated on the *x*-axis. *, *p* < 0.05 and ****, *p* < 0.0001 by one-way ANOVA with Dunnett’s multiple comparisons test vs. Alum + MPLA.

To examine whether increasing the antigen dose enhanced immune responses and, potentially, improved protection, mice were immunized with a 3-Ag combination vaccine formulation containing 10 µg of NGO0861 and 25 µg of NG01438 and NGO1802. IgG antibody levels against the individual antigens showed an increased magnitude compared to the 10 µg-dose 3-Ag vaccine (Figure 7A and Figure 5A, respectively). No shift was observed in the IgG subclasses profile (Figure 7B–D), which remained Th-2-skewed; a small, apparent increase in IgG antibodies that reacted with *N. gonorrhoeae* was observed (Figure 7E,F), and IgM antibody responses to *N. gonorrhoeae* (Alum + MPLA, ~2.5–3 µg/mL; 3-Ag vaccine, ~4.5 µg/mL) remained similar to those elicited by the 10 µg-dose 3-Ag vaccine (see Table S2A). These results suggested that antibody responses induced by the 10 µg-dose 3-Ag vaccine were already near maximal levels in the current immunization conditions and were consistent with the observation that SBA titers remained similar—~1/160 against *N. gonorrhoeae* F62 (Figure 7G) and between 1/20 and 1/40 for *N. gonorrhoeae* FA1090.

## 4. Discussion

The development of an effective vaccine against *Neisseria gonorrhoeae* remains a global public health priority, as antimicrobial resistance continues to compromise treatment options. Identification of broadly protective vaccine antigens is one of the major challenges. We have previously established the predictive value of CASS, an infection-informed discovery platform designed to prioritize proteins expressed during natural human infection with characteristics favorable for vaccine development [37,38,39]. In this study, we evaluated six new CASS targets.

Their biological characteristics illustrate the complexity of antigen selection: NGO0588 and NGO0861 have no known function, but presence of the NGO0588 meningococcal homolog, NMB1317a, within the OMV proteins pool [48] makes this protein an interesting potentially cross-protective target. NGO0694 is predicted to be an autotransporter protein [49] and may also contribute to bacterial interactions with the host. NGO0757, homolog to a Spy/CpxP family protein refolding chaperone [50], has been hypothesized to participate in P pilus formation, a process important for gonococcal infection [57]. As a putative periplasmic-associated lipoprotein implicated in protein folding and oxidative stress resistance, NGO1438 may also contribute to necessary metabolic control pathways important for bacterial survival [58]. NGO1802, a putative member of the OmpH/Skp family (major periplasmic chaperone of the “holdases” family [59]) not only may play a key role in membrane integrity, but is also homologous to the meningococcal protein NMB0181 [60], present in the serogroup B meningococcal outer membrane vaccine proteins pool [53]. The six antigens were all well conserved among gonococcal isolates, a desirable characteristic for vaccine development. Conservation is particularly important for *N. gonorrhoeae*, and genetic and antigenic variability have historically complicated vaccine design.

Each individual antigen induced a robust antigen-specific IgG antibody response directed against both the individual proteins and *N. gonorrhoeae* organisms. Both qualitative and quantitative differences were observed in the mouse antisera immunoreactivity with the targets, which could be explained by potential differences in their intrinsic immunogenicity, their expression levels, localization within the bacteria (periplasm vs. outer membrane) and antibody accessibility to immunogenic epitopes in these distinct compartments. This may explain the scarcer recognition of NGO0757 (a periplasmic protein) vs. that of antigens that are also predicted to be associated with the outer membrane (i.e., NGO0861, NGO1438 or NGO1802). It is also possible that structural, biochemical or biophysical constraints may influence the ability of the antigens to be efficiently substrate-immobilized, as in the case of purified NGO0861, which appeared less immunoreactive in vitro than in the context of whole bacteria. Other differences in the immunogenic properties of the six candidates also emerged regarding the Th-bias induced. For example, despite all proteins being adjuvanted with Alum + MPLA, some (i.e., NGO0757, NGO1802) induced stronger Th2-skewed antibody response than others (i.e., NGO1438), and NGO0588 induced a more Th1-biased response, along with differences in the cytokine profile elicited. Parsing whether these properties could contribute to protective efficacy will require further investigation. Although production of serum antibodies alone may not necessarily define the value of a vaccine candidate antigen, recognition by human sera from DGI subjects suggested that antigens are expressed in vivo and capable of eliciting a response in humans, particularly NGO1802. It is possible that immunization with a vaccine containing these antigens will induce higher antibody production. Interestingly, analysis of serum bactericidal activity of the immunized mice sera indicated that four out of the six antigens induced measurable SBA titers against both *N. gonorrhoeae* strains tested. However, the criteria for antigen selection in vaccine development require considerations beyond antigen immunogenicity and functional immune responses: ease of antigen production and proper refolding, and ability to scale up and manufacturing are important factors. Thus, despite inducing the highest SBA, NGO0588 was considered a less attractive candidate because it was insoluble. The remaining three soluble antigens, NGO0861, NGO1438, and NGO1802, were examined as a multi-antigen combination vaccine, a choice supported by the observation that combining mouse antisera against the individual antigens increased antibody functional activity. Serum IgG antibody responses to the trivalent vaccine formulation remained robust, mucosal IgG antibodies were also present (although it is not known if these contribute to protection against gonococcal colonization), and enhanced bacterial killing was observed. However, in the mouse model of gonococcal vaginal infection, the trivalent vaccine did not induce a statistically significant protection compared to the adjuvant control. The lack of concordance between enhanced serum bactericidal activity, mucosal antibody responses and infection outcomes requires further evaluation of these antigens. Other mechanisms besides SBA (the most widely used functional assays for gonococcal vaccines evaluation) should also be evaluated, i.e., opsonophagocytosis and inhibition of epithelial colonization. Indeed, no validated immune correlate of protection currently exists for gonorrhea [61]; however, although no strong correlation between SBA titers and protection has been shown for OMV vaccines (Bexsero, Ng OMV) [62], this paradigm is true for some subunit vaccines [63,64,65,66]. Statistical significance may be reached using a larger mouse cohort. It is also possible that the 3-Ag vaccine formulation can be adjusted for reaching significant protection in vivo. Our preliminary antigen dosing studies indicated that more antibodies may be produced in response to immunization with more antigen, but SBA titers remained the same. Whether immunization with a high-dose 3-Ag combination vaccine can lead to better protection in vivo remains to be determined. Indeed, increasing antibody quantity alone may not necessarily enhance the efficacy of a vaccine; the quality of the antibody responses, including affinity maturation, antibody subclass type and complement-fixing ability, is likely a more important determinant of protection. This is a general theme in vaccine research, and particularly true for gonococcal vaccines. Not all antibody responses are equally protective, and high antibody titers do not necessarily correlate with bactericidal activity or protection in vivo [20,62,67,68,69]. Adjuvant type studies and/or vaccine composition studies are needed, which may have a greater impact than the antigen dose alone and provide a better understanding of multifactorial immune responses beyond bactericidal antibodies.

## 5. Conclusions

Our work expands the portfolio of vaccine targets identified through CASS and strengthens the evidence in support of infection-informed antigen discovery for gonococcal vaccine development. Future studies focused on vaccine optimization, mechanistic correlates of protection, and evaluation of expanded antigen combinations will be important for advancing CASS candidates development.

## Figures and Tables

**Table 1:** Summary of the properties of the six CASS antigens [37].

Antigen	mRNA Expression In Vivo [37] (RPKM)		PREDICTED						Core Genome ^e^
			Antigen Score ^a^	Cell Local. ^b^	Structure ^c^			Function ^d^ Homology/Annotation	
					TM Domain	Signal Sequence	Size kDa		
	(M)	(F)							
NGO0588 (NEIS1253)	91	141	0.5	P/OM	0	Yes	16	DUF4189 domain-containing protein	Yes
NGO0694 (NEIS2653)	56	106	0.56	OM	0	-	97.9	Autotransporter, outer membrane beta-barrel domain-containing protein	Yes
NGO0757 (NEIS1146)	60	107	0.52	P	0	Yes	16.6	Spy/CpxP family protein refolding chaperone	Yes
NGO0861 (NEIS2662)	510	868	0.58	P/OM	0	Yes	9.25	Hypothetical lipoprotein	Yes
NGO1438 (NEIS0490)	95	159	0.42	P	0	Yes	28.7	DsbC family protein	Yes
NGO1802 (NEIS0172)	53	104	0.43	P/OM/IM	0	Yes	19	OmpH/Skp family outer membrane protein	Yes

^a–e^, CASS targets properties determined as described in [39] using the prediction tools: ^a^, VaxiJen (cut-off set at 0.4); ^b^, PSORTb, PredictProtein Gneg-mPLoc and Phobius; ^c^, TMHMM, SignalP, LipoP; ^d^, BLASTp, UniProtKB, PFAM, KEGG, PubMLST, Vaxign; ^e^, PubMLST. P, periplasm. OM, outer membrane. IM, inner membrane.

**Table 2:** Alleles analysis in 36,931 *N. gonorrhoeae* strains in PubMLST [42].

Locus	Total Alleles	Frequency ^a^		Distribution ^b^ (% of Total)	*p*-Distance	Polymorphic Sites ^c^	Non-Synonymous Mutations ^d^
		Allele	Strains				
*ngo0588*	80	10	20,050	52.3	0.0024	78	-
		14	3877	10.5			Q19R
		11	3018	8.2			A100V
		90	2947	8			-
		92	2852	7.7			Q19R
*ngo0694*	171	2	16,830	45.6	0.00067	506	**
		7	4775	13			-
		NV	3384	9.16			-
*ngo0757*	63	21	35,076	95	0.00017	144	-
*ngo0861*	50	1	36,446	99	0.00007	49	-
*ngo1438*	154	6	20,386	55.2	0.0016	242	-
		13	6908	18.7			-
		121	4085	11			K233Q
*ngo1802*	122	21	17,700	48	0.0048	73	I2T
		23	5162	14			-
		13	2815	7.6			I2T, T11A, T19A, N46K
		159	2780	7.5			T19A, T21A
		122	2459	6.6			-
		124	2202	6			I2T, T11A, T19A, N46K

^a^, Most frequent alleles in the highest number of strains; ^b^, % of strains expressing the high frequency alleles ^a^ in the total number of isolates available in PubMLST; ^c^, Polymorphisms in the most frequent alleles ^a^; ^d^, Amino acid changes; **, Internal stop codon mutation at residue 214. NV, no values assigned.

**Table 3:** Serum bactericidal activity (SBA).

	*N. gonorrhoeae* F62		*N. gonorrhoeae* FA1090	
Mouse Antisera	SBA Titer	% Survival	SBA Titer	% Survival
Anti-NGO0588	1/160	46 ± 20 ^a^	1/320	44 ± 7 ^b^
Anti-NGO0694	1/40	46 ± 26 ^b^	1/10	91 ± 10
Anti-NGO0757	1/10	58 ± 18	ND	ND
Anti-NGO0861	1/40	45 ± 25 ^c^	1/10	38 ± 26 ^b^
Anti-NGO1438	1/40	38 ±17 ^c^	1/10	24 ± 10 ^a^
Anti-NGO1802	1/20	32 ± 14 ^c^	1/10	42 ± 26 ^b^
Alum + MPLA	1/10	89 ± 23	1/10	96 ± 25

^a^, *p* < 0.01; ^b^, *p* < 0.0005; ^c^, *p* < 0.0001 vs. adjuvant sera by one way ANOVA with Dunnet’s multiple comparisons test. ND, not determined.

## Data Availability

The raw data supporting the conclusions of this article will be made available by the authors on request.

## Supplementary Materials

The following supporting information can be downloaded at: https://www.mdpi.com/article/10.3390/vaccines14090798/s1.

## Abbreviations

The following abbreviations are used in this manuscript:

CASS	Candidate Antigen Selection Strategy
SBA	Serum bactericidal activity
STI	Sexually transmitted infection
MPLA	Monophosphoryl Lipid A
