# Supplementary material for: Preclinical Evaluation of Novel Conserved Neisseria gonorrhoeae Vaccine Candidates Identified Through Infection-Informed Antigen Discovery
Source: Vaccines (Basel). 2026 Sep 10;14(9):798. doi: 10.3390/vaccines14090798 (PMC13611482; doi:10.3390/vaccines14090798)
Supplement: Supplementary file 1 [file vaccines-14-00798-s001.zip › vaccines-4540935-supplementary.pdf]

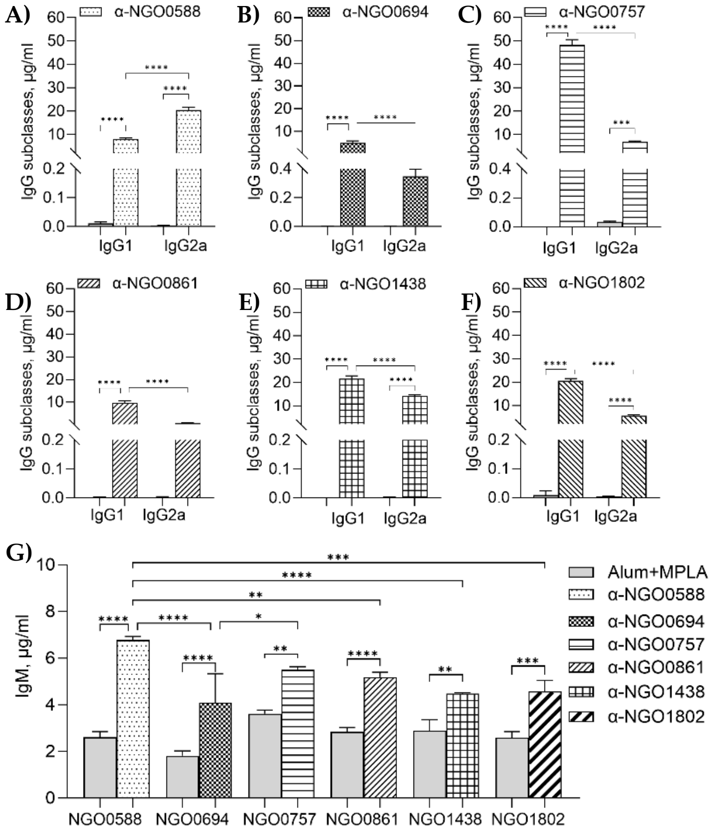

Figure S1. **Serum IgG antibody subclasses and IgM antibody ELISA against the purified antigens. A-F)** IgG1 and IgG2a antibody subclasses against each antigen in pooled sera (3rd) from mice immunized with Alum+MPLA alone (gray bars) or each antigen and Alum+MPLA (patterned bars). **G)** IgM antibodies in sera as above. Antibodies were quantified in µg/ml ± SD using a mouse IgM standard curve. * $p < 0.05$, ** $p < 0.005$, *** p , 0.0005 and **** $p < 0.0001$ by one way ANOVA with Tukey's multiple comparisons test.

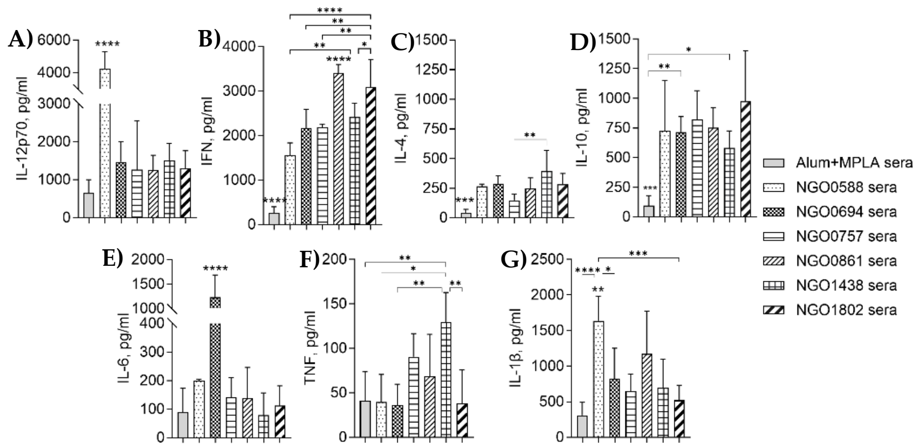

Figure S2. **Serum cytokine ELISA. A)** IL-12p70**, B)** INF-γ, **C)** IL-4**, D)** IL-10, **E)** IL-6, **F)** TNF-α and **G)** IL-1β levels in pooled sera (3rd) from mice immunized with Alum+MPLA alone (gray bars) or antigens and Alum+MPLA (patterned bars). Cytokines were expressed in pg/ml ± SD. * p < 0.05, ** p < 0.005, *** p , 0.0005 and **** p < 0.0001 by one-way ANOVA with Tukey's multiple comparisons test.

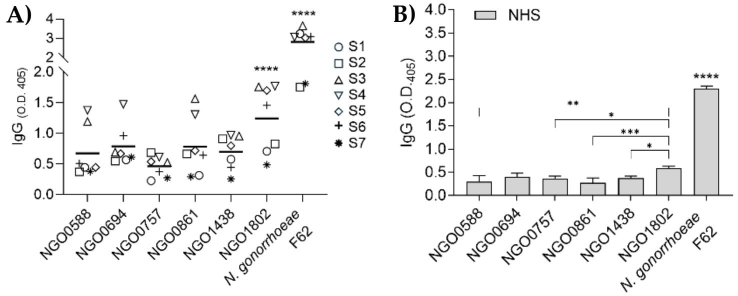

Figure S3. Human sera ELISA. Total IgG antibodies in **A)** Banked de-identified sera from women with disseminated gonococcal infection (DGI) (n=7) against each antigen and *N. gonorrhoeae* F62. Individual sera (1:100 dilution) are shown by different symbols; IgG levels are expressed as O.D.$_{405}$ minus the blank ± SD. **** $p < 0.0001$ by 2way ANOVA with Tukey's multiple comparisons test. **B)** Commercially available pooled normal human serum (NHS) as above. *, $p < 0.05$; *** $p < 0.0005$ and **** $p < 0.0001$ by one-way ANOVA with Tukey's multiple comparisons test.

Supplementary Table S1: Allele analysis in 47424 *N. meningitidis* in PubMLST [42]

Locus	Total alleles	Frequency		Distribution (% of total)	p-Distance	Polymorphic Sites	Non-Synonymous Mutations
		Allele	Strains				
nmb1317a nmc1253	326	4	12931	27.26	n/a	242	A56S, M102L, E105D
		1	9391	19.8			-
		7	5154	10.87			A101V, V132A
		8	4235	8.93			A101V
		76	3462	7.3			S146F
		16	2822	5.95			V132A
neis2653	-	-	-	-	n/a	-	-
neis1146	262	1	22200	46.8	n/a	248	-
		8	5799	12.23			-
		4	5178	10.92			P39L, S56G, T66A
		3	3832	8.08			P39L, S56G, T66A
		10	3614	7.62			P39L, S56G, T66A
neis2662	-	-	-	-	n/a	-	-
nmb0550 nmc0490	515	4	7958	16.78	n/a	579	-
		5	7154	15.08			V47A
		1	5702	12.02			S101N, V105I, R116K, E124K, G126D, F129L, E132D, I136V, D137N, K140Q, N141S, ***
		2	4091	8.63			-
		52	3121	6.58			E56K
		32	2897	6.11			-
		31	2809	5.92			D154N
		11	2756	5.81			V47A
nmb0181 nmc0172	606	1	16122	33.99	n/a	380	-
		3	9308	19.63			-
		7	7428	15.66			E95V
		9	3479	7.34			-
		4	2630	5.55			-

*** 6 amino acids insertion between residues 44 and 75; n/a: not calculated

Table S2A: Vaginal lavages IgG and serum IgM antibodies against *N. gonorrhoeae* F62 (μg/ml ± s.d.)

	Secreted IgG	Serum IgM
Alum+MPLA	0.08 ± 0.06	3.8 ± 0.4
3-Ag vaccine	0.54 ± 0.1[b]	4.9 ± 0.7

Table S2B: Vaginal lavages IgG and serum IgM antibodies against *N. gonorrhoeae* F62 (μg/ml ± s.d.)

**Individual antigens**		
Alum+MPLA	0.03 ± 0.018	1 ± 0.7
NGO0861	0.33 ± 0.18 [a]	1.27 ± 0.1
NGO1438	0.36 ± 0.15[a]	1.36 ± 0.1
NGO1802	0.44 ± 0.13 [b]	1.64 ± 0.06

[a] , $p < 0.01$ and [b] $p < 0.0005$ vs Alum+MPLA by one way ANOVA with Tukey's multiple comparisons test
